# Strategies for Lipid Production Improvement in Microalgae as a Biodiesel Feedstock

**DOI:** 10.1155/2016/8792548

**Published:** 2016-09-20

**Authors:** L. D. Zhu, Z. H. Li, E. Hiltunen

**Affiliations:** ^1^Faculty of Technology, University of Vaasa and Vaasa Energy Institute, P.O. Box 700, 65101 Vaasa, Finland; ^2^Hubei Collaborative Innovation Center for Green Transformation of Bio-Resources and Faculty of Resources and Environmental Science, Hubei University, Wuhan 430062, China

## Abstract

In response to the energy crisis, global warming, and climate changes, microalgae have received a great deal of attention as a biofuel feedstock. Due to a high lipid content in microalgal cells, microalgae present as a promising alternative source for the production of biodiesel. Environmental and culturing condition variations can alter lipid production as well as chemical compositions of microalgae. Therefore, application of the strategies to activate lipid accumulation opens the door for lipid overproduction in microalgae. Until now, many original studies regarding the approaches for enhanced microalgal lipid production have been reported in an effort to push forward the production of microalgal biodiesel. However, the current literature demonstrates fragmented information available regarding the strategies for lipid production improvement. From the systematic point of view, the review highlights the main approaches for microalgal lipid accumulation induction to expedite the application of microalgal biodiesel as an alternative to fossil diesel for sustainable environment. Of the several strategies discussed, the one that is most commonly applied is the design of nutrient (e.g., nitrogen, phosphorus, and sulfur) starvation or limitation. Other viable approaches such as light intensity, temperature, carbon dioxide, salinity stress, and metal influence can also achieve enhanced microalgal lipid production.

## 1. Introduction

Energy crisis, global warming, and climate changes have led to an ever-increasing concern on the sustainability issues of fossil fuels utilization as energy supply. Biofuels as types of renewable, alternative energy are recognized with the highest potential to satisfy the global energy demand. The biofuel feedstock mainly consists of the following sources: straw, wood materials, wood wastes, energy plants, sugarcane, manure, and many other agricultural coproducts or byproducts [1]. It is believed that biofuel production has several advantages such as reduction of country's reliance on crude oil imports, job creation, and farmers' income increase [2–4]. On the basis of feedstock differences, biofuels can be divided into three categories: the first generation, the second generation, and the third generation. First generation biofuels use edible feedstock such as soya beans, wheat, corn, rapeseed, oil crops, maize, sugarcane, and sugar beet, while second generation biofuels are derived from wastes and dedicated lignocellulosic feedstock such as switchgrass and jatropha [5]. One of the major disadvantages of both first and second generation biofuels is that the cultivation of these food or nonfood crops as biofuel feedstock might compete for limited arable farmland, which should be utilized to cultivate crops as food feedstock. Thus, biofuels are not considered renewable and sustainable if they are derived from food or nonfood crops [6]. Biofuel production from food crops grown in farmland will affect food security and prices, while the cultivation of nonfood energy crops will result in competition with food crops for farmland.

In response to the challenges outlined above, microalgae have received a global attention as a promising biofuel feedstock. Compared to first and second generation biofuel feedstock, microalgae as third generation biofuel feedstock have some distinguishable features, such as high photosynthetic efficiency, rapid growth, high lipid content, high CO_2_ mitigation efficiency, noncompetition with food crops for farmland, and less water demand than land crops [5, 7–9]. As photosynthetic organisms, microalgae are able to capture solar energy and use water and atmospheric CO_2_ to accumulate biomass in forms of organic ingredients such as lipids [10]. During the photosynthesis, neutral lipids are accumulated as triacylglycerols (TAGs) in microalgal cells [11]. Through transesterification, TAGs can be further transferred into various types of fatty acid methyl esters, the efficient compositions of biodiesel.

To generate large amount of microalgal biomass and meet the energy consumption demand, mass scale microalgal cultivation for biodiesel production is a plausible solution in the future [12]. Meanwhile, the improvement of lipid content in microalgal cells also presents a direction towards the sustainable development of microalgal biodiesel. Thus, it is extremely important to apply feasible strategies to induce microalgal lipid accumulation. The production and accumulation of microalgal lipids are found to be an indispensable buffer against the culturing conditions. Stored lipids not only ensure the survival of microalgal cells but also serve as a source of energy for cell multiplication such as nuclear division and DNA replication [11].

*Objective and Structure of This Study*. Plenty of original research articles that exactly investigate certain strategies for enhanced microalgal lipid production have been published. These strategies play crucial roles in triggering microalgal lipid accumulation. However, the current literature has not systematized all of the most promising strategies, and there is only fragmented information available. In other words, as yet, no comprehensive review has been published on those strategies for enhanced microalgal lipid production. Therefore, this review aims to bridge the gap and its objective is to systematically concentrate on the main lipid induction strategies that can evidently promote microalgal lipid production. The contribution of this review paper lies in the foundation for stakeholders, authorities, and practitioners to better understand microalgal lipid induction strategies and their significances in practice. In the coming parts, the authors first illustrate the metabolic pathways of lipid accumulation (Section 2), followed by the comprehensive discussion of approaches for lipid production improvement (Section 3). Finally, a summary of this article is concluded (Section 4).

## 2. Metabolic Pathways of Lipid Accumulation

The research on lipid-rich microalgal cultivation for biodiesel production has received an increased interest. Lipids and polyglucans are the energy and carbon reserves in microalgal cells, but polyglucans represent less concentrated stores of metabolic energy than lipids [11]. Both lipids and polyglucans not only ensure the survival of microalgal cells such as in night periods as well as in periods with variable light intensities but also supply energy for biological processes associated with the multiplication of microalgal cells, such as the replication of DNA, division of nuclear, cytokinesis, and formation and liberation of daughter cells. According to Berg et al. [13], the complete oxidation of fatty acids can generate energy at 9 kcal g^−1^ (38 kJ g^−1^), compared to around 4 kcal g^−1^ (17  kJ  g^−1^) for carbohydrates. Lipids include two types: neutral lipids that serve as the energy reserves and polar lipids that are constituents of organelles and membranes. Microalgal cells accumulate and store neutral lipids in the form of triacylglycerols (TAGs).

**Figure 1:**
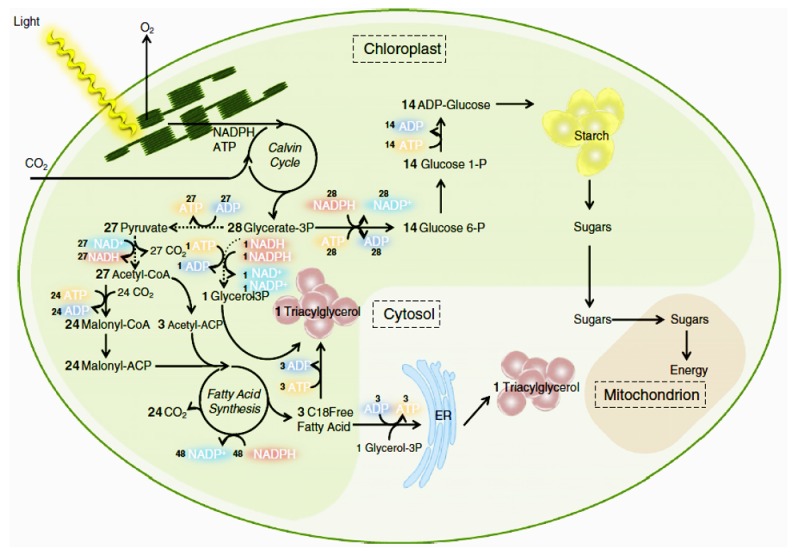
Simplified triacylglycerol (TAG) metabolism in green microalgae [14]. The dashed lines refer to the reactions that occur in the cytosol. The figure illustrates two possible pathways for TAG formation following the assumed route in the chloroplasts or over the endoplasmic reticulum (ER) membranes in the cytosol.

Microalgal cell cycle contains several consecutive procedures, including cell growth, DNA replication, nuclear division, and cellular division. Metabolism of both starch and lipid begins with an identical initial pool of molecules containing three carbons such as glyceraldehyde 3-phosphate (GAP) and 3-phosphoglycerate (3PG) [14].  Figure 1 illustrates metabolic pathways that influence the accumulation of lipids by common C3 precursors. As for autotrophic microalgae, light capture for photosynthesis is crucial for microalgal growth to accumulate energy reserves such as lipids. As a result, DNA replication and nuclear and cellular division in cell cycle can be completed through the utilization of the reserves to meet requirements of carbon and energy [15].

The formation of both TAG and starch competes for carbon via common C3 precursors, resulting in carbon partitioning. However, the mechanism behind carbon partitioning together with the switch from starch towards the production of TAG has not been completely understood in the literature. When the route towards starch formation is inhibited, the pathway towards the formation of TAG molecules is improved [16]. Li et al. [17] suggested that the starch content of starchless mutants of microalgae* C. reinhardtii* was limited or even completely absent, leading to an increased TAG content in contrast to the wild type. Despite the increase in TAG content, Li et al. [18] found that growth of starchless microalgae* C. reinhardtii* was markedly inhibited by the inserted mutation, giving rise to the decrease of TAG productivity.

## 3. Approaches to Lipid Production Improvement

The composition and quantity of lipids are species-dependent and can be affected by external cultivation conditions, such as light intensity, temperature, carbon dioxide, nutrient starvation, salinity stress, and metal stress. The overproduction or overaccumulation of lipid reserves presents an indispensable buffer against changeable external cultivation conditions.

### 3.1. Light Intensity

Microalgal growth needs the input of light during the photosynthesis. As one of the key factors, light affects the performances of microalgal growth and the compositions [5]. Adequate light intensity favors the overproduction of microalgal lipids [19]. This might be because sufficient light intensity is beneficial to the storage of excess photoassimilates, which are further converted into chemical energy [20]. Microalga* Nannochloropsis* sp. experienced the accumulation of the highest amount of lipids (47% of DW) under the conditions with the highest light intensity (700 *μ*mol photons s^−1^ m^−2^) [21]. Takeshita et al. [22] found that* C. sorokiniana*,* C. viscosa*,* C. emersonii*,* C. vulgaris*,* P. beijerinckii*, and* P. kessleri* CCALA255, NIES-2152, and NIES-2159 were able to increase the productivity of lipids under high light intensity of 600 *μ*mol photons m^−2^ s^−1^. It has been seen that the lipid content of the microalgae* Scenedesmus abundans* kept on rising as the light intensity increased from 3000 to 6000 lux [23]. The highest lipid content of 32.77% was achieved, when the culture was under the light intensity of 6000 lux, followed by 27.10 and 21.20% in the culture with 5000 and 3000 lux intensity, respectively. Another study indicated that* Botryococcus* sp. had shown the highest lipid percentage (35.9%) at 6000 lux [24]. However, Sun et al. [25] suggested that highest lipid percentage (33.0%) of microalgae* N. oleoabundans* HK-129 was achieved at 14,800 lux intensity. Thus, different microalgal species have the highest lipid content at variable light intensities, since they indicate different efficiencies in light utilization. From this point of view, the ability to use light is microalgae-specific.

Either limited or saturated light intensity cannot favor the growth of microalgae. When light intensity is fairly low, for example, below the compensation point, microalgal biomass concentration compromises, leading to poor growth and thus negatively impacting lipid accumulation [5]. After the compensation point, microalgae experience the increased growth as the light intensity increases, and the maximum photosynthetic efficiency occurs when it arrives at the light saturation point. Therefore, the positive effect of light intensity increases on lipid accumulation functions only up to a point [11]. Extremely high light intensity will cause photoinhibition, damage microalgal photosystems, and thus reduce lipid accumulation.

### 3.2. Temperature

The effect of temperature on microalgal growth and lipid production is similar to that of light intensity. Microalgal growth as well as lipid production exponentially increases to a certain extent as the temperature increases and reaches an optimal level [26]. The optimal value of temperature where the highest biomass production is achieved varies from species to species [27]. Microalgae* C. vulgaris* accumulated maximum lipids at 25°C, while the decrease of temperature resulted in an obvious decrease in the lipid content [28]. The temperature of 20°C was found to be the optimal temperature for microalgae* Scenedesmus* sp. to produce lipids [29]. The lipid concentration of microalgae* S. obliquus* ranged from 18 to 40% of DW, when the temperature ranged from 20 to 27.5°C [11]. Converti et al. [28] suggested that as the temperature increased from 20 to 25°C, the lipid content of* N. oculata* simultaneously increased from 7.9 to 14.9%. In another study, the optimum temperature of* C. minutissima* was found to be 20°C, where lipid productivity was the highest [30].

Increase of temperature to an optimal level causes the increase of total lipid content. However, it does not mean that all types of lipids experience the increase. Wei et al. [31] studied temperature effects on lipid properties of microalgae* Tetraselmis subcordiformis* and* Nannochloropsis oculata* and found that increased temperature led to a decrease in neutral lipids and polyunsaturated fatty acids but an increase in saturated and monounsaturated fatty acids. Likewise, James et al. [32] investigated temperature modulation of fatty acid profiles for microalgae* Chlamydomonas reinhardtii* and suggested that a switch to temperatures lower than 25°C could decrease the total amount of stored fatty acids but increase the content of unsaturated fatty acids.

### 3.3. Carbon Dioxide

As for phototrophic microalgae, CO_2_ ensures carbon supply for photosynthesis. Optimal growth of microalgae needs adequate amount of dissolved CO_2_. In general, as the quantity of CO_2_ increases to an optimal level, the growth of microalgae and production of lipids increase. Aeration of gas mixture with a high concentration of CO_2_ can meet the requirement for CO_2_. The amount of CO_2_ in atmospheric air is not sufficient. Li et al. [33] suggested that aeration with pure air resulted in a decrease in growth and lipid production of microalgae* Parachlorella kessleri*. Limited amount of CO_2_ available in cultures slows down the metabolism of microalgae, causing reduced lipids [34]. To reduce costs, flue gas (rich in CO_2_) can be introduced into microalgal culturing systems as a carbon source. However, high content of CO_2_ will also affect the growth of microalgae. This is because unutilized CO_2_ in culture will be converted to carbonic acid (H_2_CO_3_), reducing the pH value of the culture [27]. Therefore, to obtain enhanced biomass and lipid production requires optimal CO_2_ levels.

The optimal amount of CO_2_ varies among microalgal species. Cultivating microalgae* Chlorella vulgaris* under 8% (v/v) CO_2_, Montoya et al. [35] achieved the maximum amount of saturated fatty acids and lipid productivity of 29.5 mg L^−1^ day^−1^. Similarly, Widjaja et al. [36] reported that the growth and lipid production of* C. vulgaris* were enhanced with increased CO_2_ concentration. The microalgae* Chlamydomonas* sp. JSC4 strain produced maximum lipid content (65.3%) and productivity (169.1 mg L^−1^ day^−1^) at 4% (v/v) CO_2_ [37]. Maximum lipid content (34% wt) was achieved, when microalgae* Chlorococcum littorale* were cultivated with 5%  CO_2_ concentration [38]. In another study, Ho et al. [39] grew green microalgae* S. armatus* at CO_2_ concentration of 2% and obtained the highest lipid content of 22.4%.

### 3.4. Nutrient Starvation

Nutrient starvation or limitation is thought to be a feasible and environmentally friendly approach for the control of the cell cycle to enhance lipid productivity [40]. So far, nutrient starvation is recognized as the most successful strategy and most widely used. In an attempt to improve lipid productivity, it is important to obtain both substantial biomass yield and high lipid content of microalgal cells. In practice, algae are grown in media with sufficient nutrients in early stages to obtain higher biomass concentration as quickly as possible, while nutrient starvation is introduced in later stages for the overproduction of lipids.

Variation in macronutrients such as nitrogen, phosphorus, and sulfur in culturing media will lead to the alteration of macromolecular composition in microalgal cells. Under nutrient stress, lipid accumulation is favored, and TAG is formed as the dominant ingredient [27]. Plenty of studies have reported that most microalgal species can improve lipid accumulation and undergo transformation under nutrient stress [41–43]. Nevertheless, as for some species nutrient deficiency will not favor the accumulation of lipids. For instance, under nutrient deficiency the microalgae* Dunaliella salina* experienced lipid content decrease from 25 to 9% but carbohydrate increase from 16 to 56% [44].

Nitrogen, phosphorus, and/or sulfur starvation is widely recognized as a main lipid inducer for green microalgae species (Table 1). Ito et al. [45] reported that under nitrogen stress conditions the quantities of neutral lipids in microalgal cells were greatly increased, while amino acids were significantly reduced to 1/20 of the amount or even less. Mandal and Mallick [46] cultivated the microalgae* Scenedesmus obliquus* under phosphorus starvation and witnessed a lipid content increase from 10.0 to 29.5%. Sato et al. [47] suggested that sulfur starvation could trigger the microalgae* Chlamydomonas reinhardtii* to accumulate TAG in microalgal cells through the diversion of metabolic carbon-flow from protein to TAG synthesis. Apart from nitrogen, phosphorus, and sulfur starvation, depletion of nutrient medium during the growth of microalgal culture has also been found as another efficient starvation treatment for the production of lipids, since the starvation of all elements in nutrient media can be attained concurrently [11, 48]. For example, Přibyl et al. [49] found that the total extractable lipid content could reach up to 57.25%, when* C. vulgaris* was cultivated under nutrient depletion conditions for 7 days.

### 3.5. Salinity Stress

To resist osmotic pressure due to salinity stress, some metabolites in microalgal cells can be produced [50]. Surrounding salinity can affect the physiological and biochemical properties of microalgae. When microalgal cells are exposed to saline environment, recovery of turgor pressure, adjustment of the absorption and release of ions via cell membranes, and accumulation of osmosis-resisting matters are triggered [51, 52]. The salinity stress created inside the cells results in increment in the lipid content. Many microalgal species have been found to be subjected to the salinity stress. Yang et al. [53] applied NaCl induction with the optimal salt concentration at the late-exponential growth phase and found that the algae* Monoraphidium dybowskii* could increase total lipid content to 41.7% in a day. Pal et al. [21] investigated the effect of NaCl on the growth of* Nannochloropsis* sp. and reported that the highest total fatty acid (TFA) content of 47.0% DW and average lipid productivity of 360 mg TFA L^−1^ day^−1^ were achieved at 13 g L^−1^ NaCl. When exposed to salt pressure,* Scenedesmus* species experienced an obvious increase in lipid content [54]. Takagi et al. [55] studied the effect of salt concentration on intracellular accumulation of lipids and triacylglycerol in marine microalgae* Dunaliella* cells and found that as salt concentrations increased intracellular lipid content increased and the final lipid content could reach up to 70%. Under salinity stress,* C. vulgaris* experienced a 21.1% increase of lipid yields [56].

Cultivation of microalgae under salt stress can also limit contaminants, invasive organisms, and competing microorganisms in microalgal systems, which presents another advantage. However, too high salinity introduced can inhibit the cell growth and change the shape and structure of microalgal cells, due to the water pressure between media and cells. Thus, an optimal range for salinity level is supposed to be determined.

### 3.6. Metal Stress

Metal ions can also affect the growth of microalgae and lipid production. Ren et al. [57] studied the effects of iron, magnesium, and calcium on biomass and lipid production of heterotrophic microalgae* Scenedesmus* sp. R-16 in a dark environment and found that the total lipid content increased to 43.2, 35.0, and 47.4%, respectively. Another study showed that* Chlorella minutissima* UTEX 2341 indicated strong resistance to copper and cadmium ions under heterotrophic culture conditions, and the lipid content was significantly increased by 93.9 and 21.1%, respectively [58]. Liu et al. [59] found that the total lipid content in cultures added with 1.2 × 10^−5^ mol L^−1^ FeCl_3 _reached up to 56.6% of dry weight and was 3–7-fold higher than other media added with lower iron concentrations. Yeesang and Cheirsilp [24] cultivated microalgae* Botryococcus* spp. in cultures added with a high iron concentration (0.74 mM) and reported that the total lipid content was increased 1.4–2.5-fold. A study was carried out using* Chlorella * species under copper exposure to evaluate the metal stress on lipid contents, and it is found that higher lipid concentrations were observed in* C. protothecoides*,* C. vulgaris*, and* C. pyrenoidosa* in the presence of copper at 4.0 mg L^−1^ concentration. In addition, Li et al. [60] investigated the production of biomass and lipids by the microalgae* Chlorella protothecoides *under copper stress conditions and achieved the optimized biomass and lipid yield of 6.47 and 5.78 g L^−1^, respectively.

## 4. Conclusions

Microalgae have been praised as a promising feedstock for the production of biodiesel. Lipids, which are energy reserves in microalgal cells, are the raw materials for biodiesel conversion. To promote microalgal biodiesel production during the scale-up process, the achievement of lipid overproduction is essential, and certain appropriate strategies can help realize the goal. Among all strategies, the most efficient and widely used one is to apply nutrient starvation. Other approaches for the induction of lipid overaccumulation include light intensity, temperature, carbon dioxide, salinity stress, and metal stress. In practice, the lipid-inducing strategies can also be combined in an effort to achieve lipid production optimization. This review paper provides stakeholders, authorities, and practitioners with the foundation for better understanding microalgal lipid induction strategies and their significances in practice. There is hope that microalgae-based lipid production can be promoted through the application of various strategies.

## Figures and Tables

**Table 1:** Lipid content of microalgae under the cultivation with nutrient stress factor.

Microalgae	Stress factor	Temperature (°C)	Culture time (d)	Metabolic type	Lipid content (%)	Reference
*Chlorella vulgaris*	Nitrogen starvation	25	10	Autotrophic	53	[61]
*Chlorella vulgaris* and* Chlorococcum oleofaciens*	Nitrogen starvation	25	12	Autotrophic	35 and 40	[62]
*Monoraphidium* sp.	Nitrogen starvation	25	5	Autotrophic	44.4	[63]
*Chlorella zofingiensis*	Nitrogen starvation; phosphorus starvation	25	28	Autotrophic	65.1; 44.7	[64]
*Scenedesmus* sp.	Nitrogen starvation	25	10	Mixotrophic	31	[65]
*Chlorella zofingiensis*	Nitrogen starvation; phosphorus starvation	25	8	Mixotrophic	41.2; 42.7	[41]
*Chlorella zofingiensis*	Nitrogen and phosphorus starvation	25	8	Mixotrophic	46.2	[41]
*Ankistrodesmus falcatus*	Nitrogen starvation; phosphorus starvation	20	16	Autotrophic	34.4; 45.9	[66]
*Chlorella protothecoides*	Phosphorus starvation	28	7	Mixotrophic	32.8	[42]
*Parachlorella kessleri*	Sulfur deprivation	20	14	Autotrophic	50.7	[67]
*Chlorella lobophora*	Sulfur deprivation	20	21	Autotrophic	50.0	[68]
*Parachlorella kessleri*	Depletion of diluted nutrient media	30	4	Autotrophic	60.0	[33]
